# Adrenoreceptors and nitric oxide in the cardiovascular system

**DOI:** 10.3389/fphys.2013.00321

**Published:** 2013-11-06

**Authors:** Valeria Conti, Giusy Russomanno, Graziamaria Corbi, Viviana Izzo, Carmine Vecchione, Amelia Filippelli

**Affiliations:** ^1^Department of Medicine and Surgery, University of SalernoBaronissi, Italy; ^2^Department of Medicine and Surgery, Doctoral School of Translational and Clinical Medicine, University of SalernoBaronissi, Italy; ^3^Department of Medicine and Health Sciences, University of MoliseCampobasso, Italy; ^4^Department of Biology, University of Naples Federico IINapoli, Italy; ^5^Vascular Physiopathology Unit, IRCCS INM NeuromedPozzilli, Italy

**Keywords:** adrenoreceptors, endothelium, nebivolol, nitric oxide, vascular tone

## Abstract

Nitric Oxide (NO) is a small molecule that continues to attract much attention from the scientific community. Since its discovery, it has been evident that NO has a crucial role in the modulation of vascular tone. Moreover, NO is involved in multiple signal transduction pathways thus contributing to the regulation of many cellular functions. NO effects can be either dependent or independent on cGMP, and rely also upon several mechanisms such as the amount of NO, the compartmentalization of the enzymes responsible for its biosynthesis (NOS), and the local redox conditions. Several evidences highlighted the correlation among adrenoreceptors activity, vascular redox status and NO bioavailability. It was suggested a possible crosstalk between NO and oxidative stress hallmarks in the endothelium function and adaptation, and in sympathetic vasoconstriction control. Adrenergic vasoconstriction is a balance between a direct vasoconstrictive effect on smooth muscle and an indirect vasorelaxant action caused by α_2_- and β-adrenergic endothelial receptor-triggered NO release. An increased oxidative stress and a reduction of NO bioavailability shifts this equilibrium causing the enhanced vascular adrenergic responsiveness observed in hypertension. The activity of NOS contributes to manage the adrenergic pathway, thus supporting the idea that the endothelium might control or facilitate β-adrenergic effects on the vessels and the polymorphic variants in β_2_-receptors and NOS isoforms could influence aging, some pathological conditions and individual responses to drugs. This seems to be dependent, almost in part, on differences in the control of vascular tone exerted by NO. Given its involvement in such important mechanisms, the NO pathway is implicated in aging process and in both cardiovascular and non-cardiovascular conditions. Thus, it is essential to pinpoint NO involvement in the regulation of vascular tone for the effective clinical/therapeutic management of cardiovascular diseases (CVD).

## Introduction

Nitric Oxide (NO) is a small gaseous molecule recognized as a ubiquitous intercellular messenger modulating crucial functions including blood flow, platelet aggregation, and neural activity (Moncada, 1994).

This molecule is synthesized from L-arginine by three isoforms of Nitric Oxide Synthases (NOSs) and all of them (nNOS, Inos, and eNOS) concur to regulate the autonomic nervous system.

NO exerts its activity essentially by stimulating soluble Guanylyl Cyclase (GC) to increase the levels of the second messenger cGMP, which in turn modulates the performance of adrenergic receptors (ARs).

Recently, many studies highlighted an important role in the regulation of the vasomotor tone of the β-adrenoreceptor subtype β_3_, which, differently from the classical β_1_- and β_2_-ARs, induces a negative inotropism in the human heart (Balligand, 1999; Salazar et al., 2013).

NO effects depend, among others, on NO concentration, compartmentalization of NOS enzymes and local redox conditions of cells and tissues and, to date, many evidences collected by both *in vivo* and *in vitro* experiments suggest a crosstalk between NO, ARs and oxidative stress in the control of endothelium homeostasis, and in the sympathetic regulation of the vascular tone (Graves and Poston, 1993; Lembo et al., 2000; Selemidis et al., 2007).

The NO pathway is directly implicated in the development and progression of diseases such as hypertension and heart failure (HF) and, recently, this molecule has been considered a promising target to develop new clinical strategies against cardiovascular pathologies (Levy et al., 2009).

In addition, it is worth noting that some studies showed that polymorphisms in genes encoding for ARs and NOS enzymes could influence aging, onset and progression of cardiovascular diseases (CVD), and response to therapy (Jáchymová et al., 2001; Garovic et al., 2003).

The main focus of this review is the mechanisms underlying the interconnection between β-ARs and NO in the cardiovascular system, and the therapeutic potential of new discoveries in this field.

## NO modulates vasomotor tone by interfering with sympathetic autonomic nervous system

In 1980s the Endothelium-Derived Relaxing Factor (EDRF), discovered by Moncada, was identified as NO (Hutchinson et al., 1987; Palmer et al., 1987) and, from that moment, several studies shed light on a countless number of important roles played by this molecule which was proclaimed Science's “Molecule of the Year 1992” (Nathan, 1992, 1995; Bredt and Snyder, 1994).

Since its discovery, it was clear that NO acts as a key modulator of the vascular tone and that its vascular effects are generally mediated by Guanosine 3′,5′-cyclic MonoPhosphate (cGMP) through the activation of guanylate cyclase. In fact, several experiments using NO donors and/or cGMP analogs have shown that cGMP is a critical and multifunctional second messenger that mediates several functions in cardiac and vascular tissues as well as the etiology and pathophysiology of cardiovascular disorders (Tulis, 2008). Both neurotransmitters and hormones released from autonomic nervous system cooperate to preserve the balance between vasoconstriction and vasorelaxation and to control cardiac muscle cells function, and it is now generally accepted that NO exerts a critical role in this context. Balligand et al., which investigated the effects of NOS inhibitors in isolated neonatal and adult rat ventricular myocytes, exposed to either muscarinic or adrenergic agonists, concluded that the physiological response of the cells to both muscarinic cholinergic and β-adrenergic stimulation is mediated, at least in part, by NO production (Balligand et al., 1993).

Cardiovascular homeostasis is regulated by NO produced by all three NOS isoforms. Several studies demonstrated, both *in vivo* (Schwarz et al., 1995) and *in vitro* (Horackova et al., 1995), that NO produced by neuronal NOS (nNOS) controls catecholamines release in response to electrical adrenergic nerve stimulation. This is very important also in consideration that elevated levels of cathecolamines are associated to several pathologic conditions such as HF (Rengo et al., 2012a).

The inducible NO Synthase (iNOS) has been also involved in several aspects of cardiovascular biology such as the defence against intracellular microorganisms (Balligand and Cannon, 1997).

Moreover, endothelial cells express, in heart and vessels of a variety of species including humans, endothelial NO Synthase (eNOS), an isoform that is activated to produce NO in response to stimulation of both adrenergic and muscarinic cholinergic receptors in cardiac myocytes (Balligand et al., 1995).

Many studies demonstrated that vascular endothelial cells might also express β-adrenoceptors (Buxton et al., 1987; Molenaar et al., 1988), thus supporting the idea that the endothelium might control or facilitate β-adrenergic effects on the vessels. The main mechanism leading to increased eNOS activity in endothelial cells is calcium-dependent (Wu, 2002), but phosphorylation at several loci of the NOS proteins has been recognized as an additional pathway to induce both activation and inhibition of eNOS activity (Bauer et al., 2003; Fleming and Busse, 2003).

Both *in vivo* and *in vitro* studies suggested that the vascular endothelium might mediate β-adrenergic vasorelaxation, though not all the results presented are in agreement with each other. For instance, it was observed that rat mesenteric resistance arteries can be relaxed by NO release upon β_1_-adrenoreceptor stimulation (Graves and Poston, 1993). Priest et al. showed an involvement of NO in β-mediated vasorelaxation in large but not in small rat arteries suggesting a role of NO strictly dependent on the vascular area (Priest et al., 1997).

Ferro et al. verified that the stimulation of β_2_-adrenoreceptors led to an increase in NO, which in turn caused relaxation of Human Umbilical Vein Endothelial Cells (HUVEC). In this study, the authors provided also a comparison between β-adrenoreceptor function measured in HUVEC and the response to β-adrenergic stimuli in intact vessels, showing the importance of endothelium in maintaining vascular homeostasis (Ferro et al., 1999). In addition, Lembo et al. suggested the existence of an endothelium NO component essential for the insulin modulation of α_2_- and β-adrenergic vascular responses. An impairment of the equilibrium between endothelial and vascular smooth muscle adrenergic signaling could contribute to the increase of vascular resistance, a pivotal phenotypical trait of essential hypertension (Lembo et al., 1997).

## Role of β_3_-adrenoceptor

Emerging evidences highlighted a role played by a third β-adrenoreceptor subtype (β_3_), traditionally known as a modulator of lipolysis in adipose tissue, as a regulator of the vasomotor tone in conjunction with β_1_- and β_2_-ARs (Trochu et al., 1999).

The involvement of a β-receptor, other than classical β_1_- and β_2_-ARs, has been suggested in several experiments which used a different concentration of non-selective β-blockers (Clark and Bertholet, 1983; Doggrell, 1990; Oriowo, 1995) and preferential β_3_-AR agonists (Berlan et al., 1995).

It is now widely accepted that the vasoactive effects dependent on the stimulation of β-ARs are strongly associated with NO production and activation (Trochu et al., 1999).

Recently, many investigations focused on β_3_-ARs, which are detected in human endothelial cells and cardiac myocytes of the human heart. These receptors subtypes are highly expressed in the atrium and, in contrast to β_1_- and β_2_-ARs, are responsible of a negative cardiac inotropic effect (Moniotte et al., 2001). Moreover, on the basis of differential expression of β_3_-ARs in the human myocardium chambers, Brixius et al. found that eNOS is activated by β_3_-AR predominantly via phosphorylation in the left ventricle and through a translocation process in the atrium (Brixius et al., 2004).

The link between β_3_ AR, eNOS and vasodilation mechanism was demonstrated also in *in vivo* models; recent advances in the field were achieved by using nebivolol, a β_1_-blocker. Dessy et al. verified that nebivolol dilates human and rodent coronary resistance microarteries, and showed that this effect is sensitive to NOS inhibition and is hampered in β_3_-adrenoreceptor-deficient mice. Moreover, the authors showed proangiogenic properties of nebivolol, which are dependent by both eNOS and β_3_-adrenoreceptors (Dessy et al., 2005).

Interestingly, β_3_-AR is upregulated during cardiomyophaties in humans and this characteristic, together with its peculiar differential expression in the human myocardium, makes it an attractive target for the development of new clinical strategies against CVD (Moniotte et al., 2001).

Many studies investigated the involvement of β_3_-AR in the onset and progression of cardiovascular clinical conditions both in animal and human models, and it seems conceivable that the stimulation of β_3_-AR leads to NO-mediated protective effects in vascular beds (Dessy et al., 2004, 2005). In addition, in case of neurohormonal stress, β_3_-ARs expressed in the endothelium promote coronary perfusion through their vasodilator and pro-angiogenic effects (Balligand, 2009).

Recently, a randomized trial, named SENIORS (Study of the Effects of Nebivolol Intervention on Outcomes and Rehospitalization in Seniors with HF) was performed to investigate the effects of nebivolol on the ejection fraction in aged patients (≥70 years).

Notably, nebivolol possesses vasodilator ancillary properties, probably mediated by the endothelial L-arginine NO pathway. In particular, it was demonstrated that the favorable hemodynamic profile of nebivolol, including the lowering of blood pressure, is partially due to NO release from endothelial cells (Maffei et al., 2006). In addition, thanks to its antioxidant activity, nebivolol prevents the detrimental effect on NO bioavailability associated to oxidative stress (Ignarro, 2004).

To date, preclinical and clinical data confirm that NO benefits might due to β_3_-ARs overexpression. However, caution should be used, as the long term effects of β_3_-ARs agonists on left ventricular function in the heart have not yet been fully evaluated.

Furthermore, many studies investigated the impact of life-style changes and non-pharmacological interventions on the cardiovascular homeostasis, and convincing evidences showed a favorable role exerted by diet and caloric restriction. For example, Nisoli et al. demonstrated that caloric restriction leads to enhanced mitochondrial biogenesis, at least in part, by inducing the activation of eNOS (Nisoli et al., 2005), and Cerqueira et al. observed a time-dependent increase of eNOS activation and NO bioavailability in vascular cells conditioned with serum of caloric restricted rats (Cerqueira et al., 2012).

Also exercise training (ET), currently used in cardiac rehabilitation (CR) programs, was recognized to improve some cardiovascular outcomes by inducing NO levels to increase, even if this strongly depends on both the type and intensity of exercise (Conti et al., 2012a, 2013).

Jenkins et al. suggested that a regular physical activity in combination with dietary restriction positively influences, in a NO-dependent manner, the expression of β-AR and natriuretic peptide receptors in adipose tissues of obese rats (Jenkins et al., 2013). In addition, Calvert et al. showed that an ET-dependent stimulation of β_3_-ARs is useful to counteract myocardial ischemia-reperfusion injury by increasing NO signaling (Calvert et al., 2011).

These and many other studies remarked a crucial role played by NO in cardiovascular homeostasis.

## Adrenoreceptors stimulation, vascular redox state and nitric oxide bioavailability

Experimental *in vivo* and *in vitro* evidences suggest a crosstalk between NO, adrenoreceptors and oxidative stress in the function and adaptation of endothelium, and in the sympathetic control of the vascular tone.

Excess of ROS and/or failure of antioxidant endogenous defense may result in ROS-mediated reduction of NO bioavailability in the cells. The influence of oxidative stress on the pathway of NO biosynthesis has been extensively investigated and its effects, due to either direct quenching or impaired synthesis of NO, strongly affect the vasodilation mechanism (Förstermann and Sessa, 2012).

It has been showed that β_2_-ARs excitation increases cellular uptake of L-arginine, an eNOS substrate, and eNOS activity can be specifically stimulated by these AR subtypes in human endothelial cells. Moreover, β_2_-ARs stimulation hyperpolarizes cellular membrane, and L-NAME, a well known NOS inhibitor, may hinder this process (Wyatt et al., 2002; Queen et al., 2006).

*In vitro* experiments using endothelial cells showed an increase of β_2_-ARs-mediated eNOS phosphorylation at serine-1177, highlighting a β_2_-AR-dependent NOS activation through a Ca^2+^ insensitive mechanism (Dimmeler et al., 1999; Queen et al., 2006; Cannavo et al., 2013a,b).

Recently, Davel et al. investigated the role of oxidative stress in sympathetic-dependent contractility of human endothelium. The authors observed an increase of contractile response in β_2_-knockout (KO) mice and showed that the loss of function of these receptors in vascular tissue can induce ROS-mediated NO impairment. Administration of α1-agonist phenylephrine in the aorta of β_2_-KO mice suggested a key role played by NO in the control of vasomotor tone. In fact, the reduction of basal content of NO enhanced the vasoconstriction rate in the aorta of mice deficient for β_2_-adrenoreceptors. These experiments suggested that β_2_-ARs in vascular tissues are necessary to maintain basal levels of NO, thus concurring to modulate vascular homeostasis (Davel et al., 2012). The lack of functional β_2_-receptors led to an increase of oxidative stress in the aorta of β_2_-KO mice, and a treatment with antioxidant superoxide dismutase was sufficient to limit the vasoconstrictor response to phenylephrine. These results suggest the existence of an important link between adrenergic pathway, NO bioavailability and oxidative stress.

Indeed, several studies have showed antioxidant properties of NO, confirming the role of this molecule to counteract superoxide anions production by NADPH oxidase, the major source of superoxide in blood vessels. Several stimuli, such as oscillatory shear stress, hyperglycemia and lipid peroxidation could cause impairment in the NADPH oxidase system that, in turn, produces accumulation of ROS and reduction of NO content (Vecchione et al., 2006).

Selemidis et al. showed that prolonged exposure of human endothelial cells to NO donors, such as long-acting nitrates, induced a significant decrease of ROS via inhibition of p47phox NADPH oxidase subunit (Selemidis et al., 2007). By reducing the oxidative stress, NO donors may exert several vascular protective effects and they could be used not only for a symptomatic treatment, but also to prevent and eventually revert many aspects of CVD.

NO antioxidant properties and the ability of NO donors to counteract NADPH oxidase-dependent superoxide production are well established. However, ROS other than superoxide anions could play a role in determining redox state imbalance and the resulting detrimental effects on NO biosynthesis. Indeed, cardiovascular side effects of drugs, such as acetylsalicylic acid and other ciclooxygenase inhibitors may be due to their influence on oxidative stress hallmarks in coronary circulation, and coronary perfusion. In this context, it was suggested that those effects can be modulated by inhibition of NOS following the increase of superoxide, hydrogen peroxide and lipid peroxidation (Barudzic et al., 2013).

Further studies in the field of the antioxidant effect of adrenergic-dependent NO modulation could be essential to develop new drugs and clinical strategies to modulate oxidative stress in vascular diseases.

## Nitric oxide and cardiovascular diseases

Given its crucial role in the autonomic nervous system control, the NO pathway is directly implicated in diseases, such as hypertension and HF.

The generation of NO in the vascular endothelium ensures the maintenance of the vasodilator tone that is required for the regulation of blood flow and pressure.

Moreover, NO bioavailability plays an important role in the pathophysiology of CVD and its reduction in endothelial cells is strictly associated to endothelial dysfunction and hypertension (Lyons, 1997; Yetik-Anacak and Catravas, 2006).

The link between endothelial dysfunction and vascular diseases is well established (Rengo et al., 2013a). It is known, for instance, that impairment of endothelial function precedes atherosclerosis (Brush et al., 1992).

Stimulation of endothelial β-adrenoreceptors improves eNOS-derived NO production. The importance of such strong molecular interconnection has been recently demonstrated in several studies on nebivolol conducted in animals and in humans. Nebivolol is a third generation β-blocker used in the treatment of hypertension which induces vasodilation by increasing NO production.

Nebivolol has a distinctive profile among β-blockers, with the greatest selectivity for cardiac β_1_-ARs and the highest β_1_-/β_2_-selectivity compared with other β-blockers, and no effect on α-receptors. Moreover, nebivolol could enhance NO release and promote neoangiogenesis in cardiac tissue via stimulation of β_3_-ARs, thus reducing heart rate and blood pressure and improving systolic and diastolic function (Toblli et al., 2012).

Many studies underlined the importance of NO in the vasorelaxation mechanism of nebivolol in humans suggesting the occurrence of an additional vascular protection in hypertension. For instance, Cockcroft et al. investigated the effects of nebivolol on human forearm and demonstrated that the drug induced a potent vasodilation hampered by NO inhibitors, such as L-NAME (Cockcroft et al., 1995). Moreover, by comparing the effects of nebivolol and atenolol, another β_1_-antagonist, on the endothelial function of hypertensive patients, Tzemos et al. showed that nebivolol, differently from atenolol, was able to lower blood pressure with a concomitant reversing action on endothelial dysfunction (Tzemos et al., 2001).

The mechanism by which nebivolol acts on NO bioactivity is still unclear, but it is conceivable that the drug increases intracellular free calcium concentration by activating phospholipase C.

Studies suggested that the NO-mediated vascular effects of nebivolol may be explained considering a pharmacological cross-reactivity between serotonin 5-HT_1_ receptor and β-ARs (Fargin et al., 1998).

In addition, nebivolol exerts systemic antioxidative properties and this effect was hypothesized as an additional factor for increasing NO bioavailability. For example, nebivolol and atenolol similarly reduced blood pressure values in hypertensive patients, but oxidative stress markers, such as LDL hydroperoxides, 8-isoprostanes, ox-LDL were significantly improved only in patients treated with nebivolol (Troost et al., 2000; Fratta Pasini et al., 2005; Wojciechowski and Papademetriou, 2008).

The antioxidative property of nebivolol concurs to consider it as an optimal therapeutic presidium. In fact it was demonstrated that permanent β-ARs stimulation, typically observed during CVD, could induce an over-expression and an activation of eNOS which in turn lead to oxidative stress through superoxide anion generation and a paradoxically consequent decrease of NO bioavailability (Davel et al., 2006).

HF is another disease in which the NO pathway is recognized to have a crucial role. It is a very complex pathology characterized by cardiovascular dysfunction and also by diminished vascular NO bioavailability (Recchia et al., 1998; Sun et al., 2000; Wiemer et al., 2001; Rengo et al., 2012c).

By using down and up-regulation of all types of human NOS genes in genetically modified mice, the involvement of NO in the pathogenesis of HF has been largely investigated and many experimental studies have demonstrated that eNOS isoform plays a protective role in HF.

For example, Janssens et al. showed that overexpression of eNOS enzyme preserves cardiac function and limits cardiac remodeling in transgenic mice over expressing human eNOS enzyme (Janssens et al., 2004).

Moreover, Jones et al. demonstrated that mice over expressing eNOS displayed reduction in pulmonary edema and increase in survival without differences in ventricular morphology and function, proposing that eNOS-derived NO might exert its beneficial role by decreasing vascular resistance (Jones et al., 2003).

Vice versa, eNOS deficient mice develop severe cardiac dysfunction and remodeling after myocardial infarction (MI).

Scherrer-Crosbie et al. studied the impact of eNOS in left ventricular remodeling after MI in eNOS- KO mice, concluding that eNOS has a key role in limiting cardiac dysfunction and remodeling, in part by decreasing myocyte hypertrophy in the remote myocardium (Scherrer-Crosbie et al., 2001; Cannavo et al., 2013a).

The modulation of renin-angiotensin-aldosterone axis and adrenergic system are key elements in the therapy of several pathologies, including Alzheimer Disease (Femminella et al., 2013) and CVD, such as coronary artery diseases and HF (Marciano et al., 2012; Rengo et al., 2012b, 2013b).

It has been reported that NO is involved in beneficial effects of drugs, including statins, Angiotensin Converting Enzyme inhibitors (ACE-I), Angiotensin II Type 1 receptor blockers (ARBs) and β-blockers.

Statin therapy significantly enhances NO bioavailability in endothelial cells and exert beneficial effects in several molecular aspects of the MI, including neovascularization, LV dysfunction, interstitial fibrosis, remodeling and survival (Landmesser et al., 2004).

Both ACE-I and ARBs generate cardioprotective effects in mice with post-ischemic HF by improving left ventricular function and attenuating fibrosis and hypertrophy (Cavasin et al., 2000). It was demonstrated that in eNOS-KO mice with HF the beneficial effects of these drugs were abolished, suggesting that NO is a key regulator of the ACE-I and ARBs effects (Liu et al., 2002).

Moreover, the treatment with β-blockers improves LV systolic function and produces positive cardiac remodeling (Colucci et al., 2007).

In particular, recent studies demonstrated that the third generation β-blockers possess important additional properties besides inhibiting β-adrenoceptors. Among them, nebivolol and carvedilol enhance the bioavailability of NO by both inducing endothelial NO synthesis and preventing free radicals-mediated NO inactivation. Therefore, these drugs show advantages compared to the conventional β-antagonists (Vanhoutte and Gao, 2013).

Several studies indicated carvedilol, in addition to conventional therapy, as the preferred β-blocker in the treatment of chronic HF.

Packer et al. performed a double-blind, placebo-controlled study in 1094 patients with chronic HF, demonstrating that carvedilol considerably reduced hospitalization and mortality rates for cardiovascular causes (Packer et al., 1996).

Moreover, combined results of studies in the US Carvedilol HF Trials Program revealed that mortality was significantly lower in carvedilol than in placebo recipients (Keating and Jarvis, 2003).

Nebivolol, endowed with a significant NO-associated vasodilating effect, did not provide the same results. SENIORS trial on nebivolol effects in elderly patients with HF displayed a reduction in cardiovascular mortality, but the US Food and Drug Administration did not approve this drug for the treatment of HF because the improvement in the systolic function of patients treated with nebivolol was not as substantial as with other β-blockers (Nair et al., 2012).

There is now compelling evidence that reduced NO bioavailability due to sympathetic hyperactivity is the major contributor to endothelial dysfunction. Thus, the effects on endothelial dysfunction of the last generated vasodilating β-antagonists might have important clinical implications, particularly in patients with resistant hypertension and possibly in the treatment of HF.

## Exercise training improves nitric oxide function

ET influences cardiovascular function and endothelial homeostasis and it is recommended to treat age-associated disorders and CVD (Leosco et al., 2008; Rengo et al., 2010; Conti et al., 2012b).

ET improves the efficiency of the endogenous antioxidant system and reduces cellular oxidation rate through the stimulation of several molecular pathways (Rinaldi et al., 2006). Oxidants, and ROS, more particularly, play an important role in several physiological processes, but their overproduction is responsible for the generation of oxidative stress, that may in turn directly or indirectly damage cellular constituents, including DNA, proteins, and lipids (Ferrara et al., 2008; Conti et al., 2013).

It has been demonstrated that ET contributes to maintain the balance between ROS and antioxidant activity (Corbi et al., 2012), thus preventing oxidative stress, which is present in all stages of both vascular and non-vascular diseases (Carrizzo et al., 2013a; Puca et al., 2013).

One of the main beneficial effects of ET on the cardiovascular system is related to its ability to enhance NO production and release.

Yang et al. demonstrated that ET induces an increase of blood flow in collateral vessels of ischemic muscles and that NO inhibition abolished this effect. The authors suggested that one of the vascular adaptations induced by ET is an increase of the NO-mediated actions, which eventually culminate in the improvement of the endothelial function (Yang et al., 2008).

The endothelial function, strongly influenced by NO, may improve after exercise both in animal models and in humans; several studies in both healthy subjects and patients with impaired NO-related vasorelaxation remarked ET ability to improve vascular structure and function and endothelial homeostasis (Green et al., 2004).

Endothelial dysfunction play a fundamental role both in the onset and progression of CVD and it has been suggested that decreased NO bioavailability could definitely favors the proatherogenic endothelial cell phenotype.

Numerous studies have underlined a fundamental role played by endothelial dysfunction in both onset and progression of CVD and it has been suggested that decreased NO bioavailability could definitely favors the proatherogenic endothelial cell phenotype (Stary et al., 1994; Libby et al., 2002; Taimeh et al., 2013). CVD progression can be slowed, stopped, or even reversed by life-style interventions, including regular physical activity and these effects are often associated with an increase in NO bioavailability and NO metabolites (Rush et al., 2005; Carrizzo et al., 2013b).

Exercise-based CR is now considered a valid therapeutic approach against CVD since it reduces morbidity and mortality. Exercise benefit depends almost in part on the exercise-based increase of NO generation, which in turn improves the endothelial function (Linke et al., 2008).

Laurent et al. investigated the effects of water-based exercises in patients with stable chronic HF or coronary artery disease, and found that this type of CR was effective in increasing the basal level of plasma nitrates. Such modification may be related to an improvement of the endothelial function and may be of significance for patients' health (Laurent et al., 2009).

In recent years evidences about the relationship among ET, adrenergic system and NO, have been accumulating. Calvert et al. clearly demonstrated that exercise protects the heart by stimulating β_3_-ARs and increasing cardiac storage of NO metabolites. The authors observed an increase of NO generation and of cardiac nitrite and nitrosothiol levels in exercised mice. In addition, they remarked a critical role played by β_3_-ARs in regulating the phosphorylation (activation) of eNOS and the generation of NO in response to exercise (Calvert et al., 2011).

Due to its short half-life, it is very difficult to assess NO endothelial production in humans and all NO bioassays, albeit undoubtedly representing a practical surrogate to measure endothelial function *in vivo*, show some relevant limitations (Green et al., 2004).

As a consequence, novel strategies to unravel the molecular mechanisms influenced by NO are required. In this context, ET could be for example considered as a practical indirect approach to study NO effects in the endothelial cells (Conti et al., 2013).

## Nitric oxide syntases and adrenoreceptors genetic variability

Extensive evidence has been recently accumulated that polymorphisms in genes encoding for ARs and NO synthase enzymes might influence aging, onset and progression of CVD and therapy response.

Montesanto et al., for instance, investigated the genetic variability linked to the three enzymatic isoforms of NO synthase (nNOS, iNOS and eNOS), and observed that genetic variants of NOS genes influenced both aging phenotypes and longevity in humans. The Authors verified the presence of a correlation between nNOS and iNOS polymorphisms and longevity from one side, and between nNOS and eNOS variants with the presence, respectively, of depression symptoms and disability from the other (Montesanto et al., 2013).

In addition, increasing evidence suggests that genetic polymorphisms are responsible for different cardiovascular outcomes following the use of antihypertensive drugs. Jáchymová et al. analysed the common polymorphism Glu298Asp, located in the eNOS gene, in a group of patients with hypertension and in age-matched healthy subjects. They found that this polymorphism was associated with an insufficient response of patients to conventional therapy, thus suggesting that this genetic variant may concur to the pathogenesis of essential hypertension (Jáchymová et al., 2001).

Zhang et al. studied several eNOS polymorphisms, highlighting a significative correlation between the presence of these variants, coronary heart diseases (CHD) and HF. In particular, this study showed that patients bearing minor allele of −690 C > T polymorphism had higher risk in CHD and minor allele carriers for −922 A > G variant had higher risk in HF.

Moreover, a genotype-dependent variability in the therapy response of patients randomized to the amlodipine or to the lisinopril and clorthalidone treatments was described (Zhang et al., 2012). Indeed, minor allele carriers treated with amlodipine showed better outcomes, when compared exclusively to those treated with lisinopril, including changes in systolic and diastolic blood pressure. These pharmacogenetic data suggested that eNOS genotyping might be useful to select the most effective and safe treatment to obtain the best individual therapy response.

Previous studies have suggested that eNOS Glu298Asp polymorphism could influence NO synthesis through the expression of a protein with different susceptibility to cleavage (Tesauro et al., 2000) and the same variant has been correlated with endothelial function (Savvidou et al., 2001; Leeson et al., 2002). These results, together with other epidemiological data, suggested that eNOS polymorphisms, other than the Glu298Asp, could play a role in influencing the onset and progression of vascular diseases, including CHD, HF and hypertension (Benjafield and Morris, 2000).

Besides the pharmacogenetic effect linked to conventional drug therapies, it was showed that Glu298Asp eNOS gene polymorphism might interact with environmental and dietary factors, such as smoking and n-3 fatty acid, influencing endothelial function (Leeson et al., 2002).

In addition, also ET-associated antihypertensive effects have been reported to vary on the basis of the individual genetic background. It is worth to note that a polymorphism (−786 T > C) in the promoter region of the eNOS gene was indicated as an influencing factor of exercise beneficial effects (Augeri et al., 2009).

Some studies suggested that changes in NO synthesis contribute to a vasodilator response variability to β_2_ agonists. It was observed that forearm blood flow response to isoproterenol is impaired in men with hypercholesterolemia, a condition associated with dysfunctional NO activity (Chowienczyk et al., 1992). Moreover, the β-adrenergic vasorelaxation in the human forearm is reduced by N-monomethyl-l-arginine (L-NMMA), an inhibitor of nitric oxide synthase, confirming the crucial role played by NO in the sympathetic regulation of vascular tone (Dawes et al., 1997).

Several polymorphisms located in the gene encoding β_2_-receptors have been correlated to the difference in the expression, coupling and agonist regulation of these receptors. Polymorphisms affecting amino acids 16, 27, and 164 are the most common genetic variants and, for example, the Arg16Gly is known to predispose to agonist-induced down-regulation and desensitization of the receptors, probably concurring to the pathogenesis of asthma severity (Green et al., 1993; Reihsaus et al., 1993).

To confirm the involvement of the NO pathway in the β_2_-adrenoreceptor-dependent vasodilation control, Garovic et al. showed that the forearm blood flow response to isoproterenol was greater in Gly 16 then in Arg 16 homozygotes and that response was inhibited by L-NMMA (Garovic et al., 2003).

To date, many authors reported that adrenoreceptor (in particular, β_2_-receptors) polymorphisms are strongly associated to cardiovascular outcomes, including blood pressure, and to predisposition (Timmermann et al., 1998; Bray et al., 2000) and treatment (Johnson and Terra, 2002; McNamara et al., 2002; Taylor and Bristow, 2004) of CVD.

The studies described above confirm that analysis of patients DNA may be useful to understand sympathetic vasodilation mechanism and implication of NO pathway and to create new therapeutical strategies against CVD.

## Conclusions

The maintenance of myocardial and vascular homeostasis is one of the many diverse physiological functions mediated by NO, a versatile and nearly ubiquitous molecule that plays a key function as a signaling molecule throughout the body. An imbalance in either the production or release of this molecule is correlated to CVD such as hypertension and HF.

**Figure 1:**
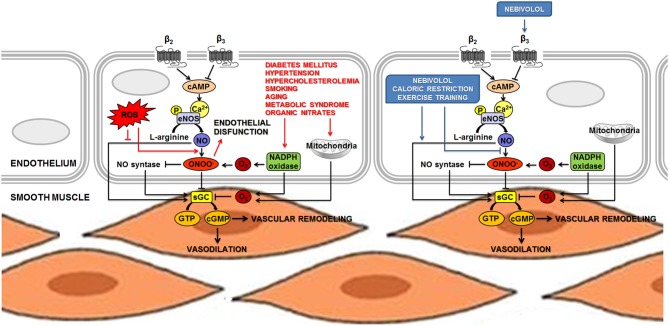
**NO is involved in the sympathetic regulation of vascular tone and in the control of endothelium homeostasis.** The main mechanism leading to increased eNOS activity in endothelial cells is calcium-dependent, but phosphorylation at several loci of the NOS proteins has been recognized as an additional pathway to induce both activation and inhibition of eNOS activity. NO diffuses to vascular smooth muscle and produces relaxation by stimulating sGC to increase the levels of the second messenger cGMP. Vascular endothelial cells might also express β-adrenoceptors, thus supporting the hypothesis that the endothelium might control or facilitate β-adrenergic effects on the vessels. Acute β-adrenergic activation caused by β-adrenoceptor agonists stimulates eNOS activity and could increase release of endothelial NO. Permanently high catecholamine levels could lead to overactivation of β-adrenoceptors, increasing eNOS activity and expression. This condition may lead to the uncoupling of eNOS, which produces O_2−_ and ONOO^−^ (ROS). An unbalanced production of NO and O_2−_ is responsible for the formation of ONOO^−^, thus provoking vascular dysfunction. Several stimuli, such as oscillatory shear stress, hyperglycemia and lipid peroxidation could cause impairment in the NADPH oxidase system that, in turn, produces accumulation of ROS and reduction of NO content. Nebivolol, a β-blocker with a distinctive profile, combines the properties of a β_1_-AR antagonist and β_3_-AR agonist. Nebivolol could enhance NO release via stimulation of β_3_-ARs and, thanks to its antioxidant activity, it prevents the detrimental effect on NO bioavailability associated to oxidative stress. Life-style changes and non-pharmacological interventions (such as caloric restriction and exercise training) show a positive role on the maintenance of cardiovascular homeostasis, at least in part, by inducing the activation of eNOS and increasing NO bioavailability. Abbreviations: AR, adrenoreceptor; cGMP, cyclic guanosine monophosphate; eNOS, endothelial nitric oxide synthase; GTP, guanosine 5′-triphosphate; NADPH, reduced nicotinamide adenine dinucleotide phosphate; NO, nitric oxide; O^−^_2_, superoxide; ONOO^−^, peroxynitrite; P, phosphoryl; ROS, reactive oxygen species; sGC, soluble guanylate cyclase. ↓ Activation; ⊥ inhibition.

It is now evident that the control of endothelium homeostasis and the sympathetic regulation of the vascular tone are the result of a complex crosstalk between NO, β-adrenoreceptors (in particular the β_3_ subtype) and oxidative stress. As an example, recent advances on the β_1_-selective antagonist nebivolol remarked the importance of NO bioavailability in the maintenance of myocardial and vascular homeostasis. A scheme representing some of the functions in which the pathway of NO is strongly involved was presented in Figure 1.

It is our opinion that the effective clinical/therapeutic management of CVD requires the understanding of the molecular determinants responsible for this crosstalk to identify new targets and develop new clinical strategies.

### Conflict of interest statement

The authors declare that the research was conducted in the absence of any commercial or financial relationships that could be construed as a potential conflict of interest.

## References

[B1] Augeri A. L. Tsongalis G. J. Van Heest J. L. Maresh C. M. Thompson P. D. Pescatello L. S. (2009). The endothelial nitric oxide synthase -786 T>C polymorphism and the exercise-induced blood pressure and nitric oxide responses among men with elevated blood pressure. Atherosclerosis 204, e28–e34 10.1016/j.atherosclerosis.2008.12.015 19155013

[B2] Balligand J. L. (1999). Regulation of cardiac beta-adrenergic response by nitric oxide. Cardiovasc. Res. 43, 607–620 10.1016/S0008-6363(99)00163-7 10690332

[B3] Balligand J. L. (2009). beta(3)-Adrenoceptor stimulation on top of beta(1)-adrenoceptor blockade “Stop or Encore?.” J. Am. Coll. Cardiol. 53, 1539–1542 10.1016/j.jacc.2009.01.048 19389565

[B4] Balligand J. L. Cannon P. J. (1997). Nitric oxide synthases and cardiac muscle. Autocrine and paracrine influences. Arterioscler. Thromb. Vasc. Biol. 17, 1846–1858 10.1161/01.ATV.17.10.1846 9351345

[B5] Balligand J. L. Kelly R. A. Marsden P. A. Smith T. W. Michel T. (1993). Control of cardiac muscle cell function by an endogenous nitric oxide signaling system. Proc. Natl. Acad. Sci. U.S.A. 90, 347–351 10.1073/pnas.90.1.347 7678347 PMC45657

[B6] Balligand J. L. Kobzik L. Han X. Kaye D. M. Belhassen L. O'Hara D. S. (1995). Nitric oxide-dependent parasympathetic signaling is due to activation of constitutive endothelial (type III) nitric oxide synthase in cardiac myocytes. J. Biol. Chem. 270, 14582–14586 10.1074/jbc.270.24.14582 7540173

[B7] Barudzic N. Turjacanin-Pantelic D. Zivkovic V. Selakovic D. Srejovic I. Jakovljevic J. (2013). The effects of cyclooxygenase and nitric oxide synthase inhibition on oxidative stress in isolated rat heart. Mol. Cell. Biochem. 381, 301–11 10.1007/s11010-013-1712-9 23749198

[B8] Bauer P. M. Fulton D. Boo Y. C. Sorescu G. P. Kemp B. E. Jo H. (2003). Compensatory phosphorylation and protein-protein interactions revealed by loss of function and gain of function mutants of multiple serine phosphorylation sites in endothelial nitric-oxide synthase. J. Biol. Chem. 278, 14841–14849 10.1074/jbc.M211926200 12591925

[B9] Benjafield A. V. Morris B. J. (2000). Association analyses of endothelial nitric oxide synthase gene polymorphisms in essential hypertension. Am. J. Hypertens. 13, 994–998 10.1016/S0895-7061(00)00282-X 10981549

[B10] Berlan M. Galitzky J. Montastruc J. L. (1995). Beta 3-adrenoceptors in the cardiovascular system. Fundam. Clin. Pharmacol. 9, 234–239 10.1111/j.1472-8206.1995.tb00290.x 7557818

[B11] Bray M. S. Krushkal J. Li L. Ferrell R. Kardia S. Sing C. F. (2000). Positional genomic analysis identifies the beta(2)-adrenergic receptor gene as a susceptibility locus for human hypertension. Circulation 101, 2877–2882 10.1161/01.CIR.101.25.2877 10869257

[B12] Bredt D. S. Snyder S. H. (1994). Nitric oxide, a physiological messenger molecule. Annu. Rev.Biochem. 63, 175–195 10.1146/annurev.bi.63.070194.001135 7526779

[B13] Brixius K. Bloch W. Pott C. Napp A. Krahwinkel A. Ziskoven C. (2004). Mechanisms of beta 3-adrenoceptor-induced eNOS activation in right atrial and left ventricular human myocardium. Br. J. Pharmacol. 143, 1014–1022 10.1038/sj.bjp.0705983 15466444 PMC1575956

[B14] Brush J. E. Jr. Faxon D. P. Salmon S. Jacobs A. K. Ryan T. J. (1992). Abnormal endothelium-dependent coronary vasomotion in hypertensive patients. J. Am. Coll. Cardiol. 9, 809–815 10.1016/0735-1097(92)90522-O 1545076

[B15] Buxton B. F. Jones C. R. Molenaar P. Summers R. J. (1987). Characterization and autoradiographic localization of beta-adrenoceptor subtypes in human cardiac tissues. Br. J. Pharmacol. 92, 299–310 10.1111/j.1476-5381.1987.tb11324.x 2823947 PMC1853669

[B16] Calvert J. W. Condit M. E. Aragón J. P. Nicholson C. K. Moody B. F. Hood R. L. (2011). Exercise protects against myocardial ischemia-reperfusion injury via stimulation of β(3)-adrenergic receptors and increased nitric oxide signaling: role of nitrite and nitrosothiols. Circ. Res. 108, 1448–1458 10.1161/CIRCRESAHA.111.241117 21527738 PMC3140870

[B17] Cannavo A. Rengo G. Liccardo D. Pagano G. Zincarelli C. De Angelis M. C. (2013a). β1-Adrenergic Receptor and Sphingosine-1-Phosphate Receptor 1 (S1PR1) Reciprocal Downregulation Influences Cardiac Hypertrophic Response and Progression to Heart Failure: Protective Role of S1PR1 Cardiac Gene Therapy. Circulation 128, 1612–1622 10.1161/CIRCULATIONAHA.113.002659 23969695 PMC3952877

[B18] Cannavo A. Rengo G. Liccardo D. Pironti G. Scimia M. C. Scudiero L. (2013b). Prothymosin alpha protects cardiomyocytes against ischemia-induced apoptosis via preservation of Akt activation. Apoptosis 18, 1252–1261 10.1007/s10495-013-0876-9 23857453

[B19] Carrizzo A. Forte M. Damato A. Trimarco V. Salzano F. Bartolo M. (2013a). Antioxidant effects of resveratrol in cardiovascular, cerebral and metabolic diseases. Food Chem. Toxicol. 62, 359–366 10.1016/j.fct.2013.07.021 23872128

[B20] Carrizzo A. Puca A. Damato A. Marino M. Franco E. Pompeo F. (2013b). Resveratrol improves vascular function in patients with hypertension and dyslipidemia by modulating NO metabolism. Hypertension 62, 359–366 10.1161/HYPERTENSIONAHA.111.01009 23753407

[B21] Cavasin M. A. Yang X. P. Liu Y. H. Mehta D. Karumanchi R. Bulagannawar M. (2000). Effects of ACE inhibitor, AT1 antagonist, and combined treatment in mice with heart failure. J. Cardiovasc. Pharmacol. 36, 472–480 10.1097/00005344-200010000-00009 11026648

[B22] Cerqueira F. M. Brandizzi L. I. Cunha F. M. Laurindo F. R. Kowaltowski A. J. (2012). Serum from calorie-restricted rats activates vascular cell eNOS through enhanced insulin signaling mediated by adiponectin. PLoS ONE 7:e31155 10.1371/journal.pone.0031155 22319612 PMC3271099

[B23] Chowienczyk P. J. Watts G. F. Cockcroft J. R. Ritter J. M. (1992). Impaired endothelium-dependent vasodilation of forearm resistance vessels in hypercholesterolaemia. Lancet 340, 1430–1432 10.1016/0140-6736(92)92621-L 1360559

[B24] Clark B. J. Bertholet A. (1983). Effects of pindolol on vascular smooth muscle. Gen. Pharmacol. 14, 117–119 10.1016/0306-3623(83)90078-2 6826021

[B25] Cockcroft J. R. Chowienczyk P. J. Brett S. E. Chen C. P. Dupont A. G. Van Nueten L. (1995). Nebivolol vasodilates human forearm vasculature: evidence for an L-arginine/NO-dependent mechanism. J. Pharmacol. Exp. Ther. 274, 1067–1071 7562470

[B26] Colucci W. S. Kolias T. J. Adams K. F. Armstrong W. F. Ghali J. K. Gottlieb S. S., (2007). Metoprolol reverses left ventricular remodeling in patients with asymptomatic systolic dysfunction: the REversal of VEntricular Remodeling with Toprol-XL (REVERT) trial. Circulation 116, 49–56 10.1161/CIRCULATIONAHA.106.666016 17576868

[B27] Conti V. Corbi G. Russomanno G. Simeon V. Ferrara N. Filippelli W. (2012a). Oxidative stress effects on endothelial cells treated with different athletes' sera. Med. Sci. Sports Exerc. 44, 39–49 10.1249/MSS.0b013e318227f69c 21659898

[B28] Conti V. Russomanno G. Corbi G. Filippelli A. (2012b). Exercise training in aging and diseases. Transl. Med. UniSa. 3, 74–80 23905056 PMC3728785

[B29] Conti V. Russomanno G. Corbi G. Guerra G. Grasso C. Filippelli W. (2013). Aerobic training workload affects human endothelial cells redox homeostasis. Med. Sci. Sports Exerc. 45, 644–653 10.1249/MSS.0b013e318279fb59 23135374

[B30] Corbi G. Conti V. Russomanno G. Rengo G. Vitulli P. Ciccarelli A. L. (2012). Is physical activity able to modify oxidative damage in cardiovascular aging? Oxid. Med. Cell. Longev. 2012, 728547 10.1155/2012/728547 23029599 PMC3458405

[B31] Davel A. P. Ceravolo G. S. Wenceslau C. F. Carvalho M. H. Brum P. C. Rossoni L. V. (2012). Increased vascular contractility and oxidative stress in β2-adrenoceptor knockout mice: the role of NADPH oxidase. J. Vasc. Res. 49, 342–352 10.1159/000337486 22627472

[B32] Davel A. P. Kawamoto E. M. Scavone C. Vassallo D. V. Rossoni L. V. (2006). Changes in vascular reactivity following administration of isoproterenol for 1 week: a role for endothelial modulation. Br. J. Pharmacol. 148, 629–639 10.1038/sj.bjp.0706749 16702995 PMC1751879

[B33] Dawes M. Chowienczyk P. J. Ritter J. M. (1997). Effects of inhibition of the L-arginine/nitric oxide pathway on vasodilation caused by beta-adrenergic agonists in human forearm. Circulation 95, 2293–2297 10.1161/01.CIR.95.9.2293 9142007

[B34] Dessy C. Moniotte S. Ghisdal P. Havaux X. Noirhomme P. Balligand J. L. (2004). Endothelial beta3-adrenoceptors mediate vasorelaxation of human coronary microarteries through nitric oxide and endothelium-dependent hyperpolarization. Circulation 110, 948–954 10.1161/01.CIR.0000139331.85766.AF 15302798

[B35] Dessy C. Saliez J. Ghisdal P. Daneau G. Lobysheva I. I. Frérart F. (2005). Endothelial beta3-adrenoreceptors mediate nitric oxide-dependent vasorelaxation of coronary microvessels in response to the third-generation beta-blocker nebivolol. Circulation 112, 1198–1205 10.1161/CIRCULATIONAHA.104.532960 16116070

[B36] Dimmeler S. Fleming I. Fisslthaler B. Hermann C. Busse R. Zeiher A. M. (1999). Activation of nitric oxide synthase in endothelial cells by Akt-dependent phosphorylation. Nature 399, 601–605 10.1038/21224 10376603

[B37] Doggrell S. A. (1990). Relaxant and beta 2-adrenoceptor blocking activities of (+/-)-, (+)- and (-)-pindolol on the rat isolated aorta. J. Pharm. Pharmacol. 42, 444–446 10.1111/j.2042-7158.1990.tb06590.x 1979630

[B38] Fargin A. Raymond J. R. Lohse M. J. Kobilka B. K. Caron M. G. Lefkowitz R. J. (1998). The genomic clone G-21 which resembles a beta-adrenergic receptor sequence encodes the 5-HT1A receptor. Nature 335, 358–360 10.1038/335358a0 3138543

[B39] Femminella G. D. Rengo G. Pagano G. de Lucia C. Komici K. Parisi V. (2013). β-adrenergic receptors and G protein-coupled receptor kinase-2 in Alzheimer's disease: a new paradigm for prognosis and therapy? J. Alzheimers Dis. 34, 341–347 10.3233/JAD-121813 23207488

[B40] Ferrara N. Rinaldi B. Corbi G. Conti V. Stiuso P. Boccuti S. (2008). Exercise training promotes SIRT1 activity in aged rats. Rejuvenation Res. 11, 139–150 10.1089/rej.2007.0576 18069916

[B41] Ferro A. Queen L. R. Priest R. M. Xu B. Ritter J. M. Poston L. (1999). Activation of nitric oxide synthase by beta 2-adrenoceptors in human umbilical vein endothelium *in vitro*. Br. J. Pharmacol. 126, 1872–1880 10.1038/sj.bjp.0702512 10372832 PMC1565965

[B42] Fleming I. Busse R. (2003). Molecular mechanisms involved in the regulation of the endothelial nitric oxide synthase. Am. J. Physiol. Regul. Integr. Comp. Physiol. 284, R1–R12 12482742 10.1152/ajpregu.00323.2002

[B43] Förstermann U. Sessa W. C. (2012). Nitric oxide synthases: regulation and function. Eur. Heart. J. 33, 829–837 10.1093/eurheartj/ehr304 21890489 PMC3345541

[B44] Fratta Pasini A. Garbin U. Nava M. C. Stranieri C. Davoli A. Sawamura T. (2005). Nebivolol decreases oxidative stress in essential hypertensive patients and increases nitric oxide by reducing its oxidative inactivation. J. Hypertens. 23, 589–596 10.1097/01.hjh.0000160216.86597.ff 15716701

[B45] Garovic V. D. Joyner M. J. Dietz N. M. Boerwinkle E. Turner S. T. (2003). Beta(2)-adrenergic receptor polymorphism and nitric oxide-dependent forearm blood flow responses to isoproterenol in humans. J. Physiol. 546(Pt 2), 583–589 10.1113/jphysiol.2002.031138 12527744 PMC2342525

[B46] Graves J. Poston L. (1993). Beta-adrenoceptor agonist mediated relaxation of rat isolated resistance arteries: a role for the endothelium and nitric oxide. Br. J. Pharmacol. 108, 631–637 10.1111/j.1476-5381.1993.tb12853.x 8096781 PMC1908020

[B47] Green D. J. Maiorana A. O'Driscoll G. Taylor R. (2004). Effect of exercise training on endothelium-derived nitric oxide function in humans. J. Physiol. 561(Pt 1), 1–25 10.1113/jphysiol.2004.068197 15375191 PMC1665322

[B48] Green S. A. Cole G. Jacinto M. Innis M. Liggett S. B. (1993). A polymorphism of the human beta 2-adrenergic receptor within the fourth transmembrane domain alters ligand binding and functional properties of the receptor. J. Biol. Chem. 268(31), 23116–23121 7901205

[B49] Horackova M. Armour J. A. Hopkins D. A. Huang M. H. (1995). Nitric oxide modulates signaling between cultured adult peripheral cardiac neurons and cardiomyocytes. Am. J. Physiol. 269(Pt 1), C504–C510 7653533 10.1152/ajpcell.1995.269.2.C504

[B50] Hutchinson P. J. Palmer R. M. Moncada S. (1987). Comparative pharmacology of EDRF and nitric oxide on vascular strips. Eur. J. Pharmacol. 141, 445–451 10.1016/0014-2999(87)90563-2 3499329

[B51] Ignarro L. J. (2004). Experimental evidences of nitric oxide-dependent vasodilatory activity of nebivolol, a third-generation beta-blocker. Blood Press. Suppl. 1, 2–16 10.1080/08038020410016557 15587107

[B52] Jáchymová M. Horký K. Bultas J. Kozich V. Jindra A. Peleska J. (2001). Association of the Glu298Asp polymorphism in the endothelial nitric oxide synthase gene with essential hypertension resistant to conventional therapy. Biochem. Biophys. Res. Commun. 284, 426–430 10.1006/bbrc.2001.5007 11394896

[B53] Janssens S. Pokreisz P. Schoonjans L. Pellens M. Vermeersch P. Tjwa M. (2004). Cardiomyocyte-specific overexpression of nitric oxide synthase 3 improves left ventricular performance and reduces compensatory hypertrophy after myocardial infarction. Circ. Res. 94, 1256–1262 10.1161/01.RES.0000126497.38281.23 15044322

[B54] Jenkins N. T. Padilla J. Rector S. Laughlin M. H. (2013). Influence of regular physical activity and caloric restriction on β-adrenergic and natriuretic peptide receptor expression in retroperitoneal adipose tissue of OLETF rats. Exp. Physiol. [Epub ahead of print]. 10.1113/expphysiol.2013.074658 23833052 PMC3839103

[B55] Johnson J. A. Terra S. G. (2002). Beta-adrenergic receptor polymorphisms: cardiovascular disease associations and pharmacogenetics. Pharm. Res. 19, 1779–1787 10.1023/A:1021477021102 12523655

[B56] Jones S. P. Greer J. J. van Haperen R. Duncker D. J. de Crom R. Lefer D. J. (2003). Endothelial nitric oxide synthase overexpression attenuates congestive heart failure in mice. Proc. Natl. Acad. Sci. U.S.A. 100, 4891–4896 10.1073/pnas.0837428100 12676984 PMC153651

[B57] Keating G. M. Jarvis B. (2003). Carvedilol: a review of its use in chronic heart failure. Drugs. 63, 1697–1741 10.2165/00003495-200363160-00006 12904089

[B58] Landmesser U. Engberding N. Bahlmann F. H. Schaefer A. Wiencke A. Heineke A. (2004). Statin-induced improvement of endothelial progenitor cell mobilization, myocardial neovascularization, left ventricular function, and survival after experimental myocardial infarction requires endothelial nitric oxide synthase. Circulation 110, 1933–1939 10.1161/01.CIR.0000143232.67642.7A 15466656

[B59] Laurent M. Daline T. Malika B. Fawzi O. Philippe V. Benoit D. (2009). Training-induced increase in nitric oxide metabolites in chronic heart failure and coronary artery disease: an extra benefit of water-based exercises? Eur. J. Cardiovasc. Prev. Rehabil. 16, 215–221 10.1097/HJR.0b013e3283292fcf 19276981

[B60] Leeson C. P. Hingorani A. D. Mullen M. J. Jeerooburkhan N. Kattenhorn M. Cole T. J. (2002). Glu298Asp endothelial nitric oxide synthase gene polymorphism interacts with environmental and dietary factors to influence endothelial function. Circ. Res. 90, 1153–1158 10.1161/01.RES.0000020562.07492.D4 12065317

[B61] Lembo G. Iaccarino G. Vecchione C. Barbato E. Izzo R. Fontana D. (1997). Insulin modulation of an endothelial nitric oxide component present in the alpha2- and beta-adrenergic responses in human forearm. J. Clin. Invest. 100, 2007–2014 10.1172/JCI119732 9329964 PMC508390

[B62] Lembo G. Vecchione C. Izzo R. Fratta L. Fontana D. Marino G. (2000). Noradrenergic vascular hyper-responsiveness in human hypertension is dependent on oxygen free radical impairment of nitric oxide activity. Circulation. 102, 552–557 10.1161/01.CIR.102.5.552 10920068

[B63] Leosco D. Rengo G. Iaccarino G. Golino L. Marchese M. Fortunato F. (2008). Exercise promotes angiogenesis and improves beta-adrenergic receptor signalling in the post-ischaemic failing rat heart. Cardiovasc. Res. 78, 385–394 10.1093/cvr/cvm109 18093988

[B64] Levy A. S. Chung J. C. Kroetsch J. T. Rush J. W. (2009). Nitric oxide and coronary vascular endothelium adaptations in hypertension. Vasc. Health Risk Manag. 5, 1075–1087 20057900 10.2147/vhrm.s7464 PMC2801631

[B65] Libby P. Ridker P. M. Maseri A. (2002). Inflammation and atherosclerosis. Circulation 105, 1135–1143 10.1161/hc0902.104353 11877368

[B66] Linke A. Erbs S. Hambrecht R. (2008). Effects of exercise training upon endothelial function in patients with cardiovascular disease. Front. Biosci. 13, 424–432 10.2741/2689 17981557

[B67] Liu Y. H. Xu J. Yang X. P. Yang F. Shesely E. Carretero O. A. (2002). Effect of ACE inhibitors and angiotensin II type 1 receptor antagonists on endothelial NO synthase knockout mice with heart failure. Hypertension. 39(Pt 2):375–381 10.1161/hy02t2.102796 11882576

[B68] Lyons D. (1997). Impairment and restoration of nitric oxide-dependent vasodilation in cardiovascular disease. Int. J. Cardiol. 62(Suppl. 2), S101–S109 10.1016/S0167-5273(97)00247-7 9488201

[B69] Maffei A. Vecchione C. Aretini A. Poulet R. Bettarini U. Gentile M. T. (2006). Characterization of nitric oxide release by nebivolol and its metabolites. Am. J. Hypertens. 19, 579–586 10.1016/j.amjhyper.2005.09.021 16733229

[B70] Marciano C. Galderisi M. Gargiulo P. Acampa W. D'Amore C. Esposito R. (2012). Effects of type 2 diabetes mellitus on coronary microvascular function and myocardial perfusion in patients without obstructive coronary artery disease. Eur. J. Nucl. Med. Mol. Imaging. 39, 1199–1206 10.1007/s00259-012-2117-9 22526959

[B71] McNamara D. M. MacGowan G. A. London B. (2002). Clinical importance of beta-adrenoceptor polymorphisms in cardiovascular disease. Am. J. Pharmacogenomics 2, 73–78 10.2165/00129785-200202020-00001 12083943

[B72] Molenaar P. Malta E. Jones C. R. Buxton B. F. Summers R. J. (1988). Autoradiographic localization and function of beta-adrenoceptors on the human internal mammary artery and saphenous vein. Br. J. Pharmacol. 95, 225–233 10.1111/j.1476-5381.1988.tb16568.x 2851349 PMC1854117

[B73] Moncada S. (1994). Nitric oxide. J. Hypertens. Suppl. 12, S35–S39 7539493

[B74] Moniotte S. Kobzik L. Feron O. Trochu J. N. Gauthier C. Balligand J. L. (2001). Upregulation of beta(3)-adrenoceptors and altered contractile response to inotropic amines in human failing myocardium. Circulation 103, 1649–1655 10.1161/01.CIR.103.12.1649 11273992

[B75] Montesanto A. Crocco P. Tallaro F. Pisani F. Mazzei B. Mari V. (2013). Common polymorphisms in nitric oxide synthase (NOS) genes influence quality of aging and longevity in humans. Biogerontology 14, 177–186 10.1007/s10522-013-9421-z 23572278

[B76] Nair A. P. Timoh T. Fuster V. (2012). Contemporary medical management of systolic heart failure. Circ. J. 76, 268–277 10.1253/circj.CJ-11-1424 22240600

[B77] Nathan C. (1992). Nitric oxide as a secretory product of mammalian cells. FASEB J. 6, 3051–3064 1381691

[B78] Nathan C. (1995). Natural resistance and nitric oxide. Cell. 82, 873–876 10.1016/0092-8674(95)90019-5 7553846

[B79] Nisoli E. Tonello C. Cardile A. Cozzi V. Bracale R. Tedesco L. (2005). Calorie restriction promotes mitochondrial biogenesis by inducing the expression of eNOS. Science 310, 314–317 10.1126/science.1117728 16224023

[B80] Oriowo M. A. (1995). Different atypical beta-adrenoceptors mediate isoprenaline-induced relaxation in vascular and non-vascular smooth muscles. Life Sci. 56, PL269–PL275 10.1016/0024-3205(95)00076-3 8614236

[B81] Packer M. Bristow M. R. Cohn J. N. Colucci W. S. Fowler M. B. Gilbert E. M. (1996). The effect of carvedilol on morbidity and mortality in patients with chronic heart failure. U.S. Carvedilol Heart Failure Study Group. N. Engl. J. Med. 334, 1349–1355 10.1056/NEJM199605233342101 8614419

[B82] Palmer R. M. Ferrige A. G. Moncada S. (1987). Nitric oxide release accounts for the biological activity of endothelium-derived relaxing factor. Nature 327, 524–526 10.1038/327524a0 3495737

[B83] Priest R. M. Hucks D. Ward J. P. (1997). Noradrenaline, beta-adrenoceptor mediated vasorelaxation and nitric oxide in large and small pulmonary arteries of the rat. Br. J. Pharmacol. 122, 1375–1384 10.1038/sj.bjp.0701528 9421285 PMC1565086

[B84] Puca A. A. Carrizzo A. Villa F. Ferrario A. Casaburo M. Maciąg A. (2013). Vascular ageing: the role of oxidative stress. Int. J. Biochem. Cell. Biol. 45, 556–559 10.1016/j.biocel.2012.12.024 23305730

[B85] Queen L. R. Ji Y. Xu B. Young L. Yao K. Wyatt A. W. (2006). Mechanisms underlying beta2-adrenoceptor-mediated nitric oxide generation by human umbilical vein endothelial cells. J. Physiol. 576(Pt 2), 585–594 10.1113/jphysiol.2006.115998 16873402 PMC1890348

[B86] Recchia F. A. McConnell P. I. Bernstein R. D. Vogel T. R. Xu X. Hintze T. H. (1998). Reduced nitric oxide production and altered myocardial metabolism during the decompensation of pacing-induced heart failure in the conscious dog. Circ. Res. 83, 969–979 10.1161/01.RES.83.10.969 9815144

[B87] Reihsaus E. Innis M. MacIntyre N. Liggett S. B. (1993). Mutations in the gene encoding for the beta 2-adrenergic receptor in normal and asthmatic subjects. Am. J. Respir. Cell. Mol. Biol. 8, 334–339 10.1165/ajrcmb/8.3.334 8383511

[B88] Rengo G. Cannavo A. Liccardo D. Zincarelli C. de Lucia C. Pagano G. (2013a). Vascular endothelial growth factor blockade prevents the beneficial effects of β-blocker therapy on cardiac function, angiogenesis and remodeling in heart failure. Circ. Heart. Fail. [Epub ahead of print]. 10.1161/CIRCHEARTFAILURE.113.000329 24029661

[B89] Rengo G. Pagano G. Squizzato A. Moja L. Femminella G. D. de Lucia C. (2013b). Oral anticoagulation therapy in heart failure patients in sinus rhythm: a systematic review and meta-analysis. PLoS ONE 8:e52952 10.1371/journal.pone.0052952 23301006 PMC3534653

[B90] Rengo G. Leosco D. Zincarelli C. Marchese M. Corbi G. Liccardo D. (2010). Adrenal GRK2 lowering is an underlying mechanism for the beneficial sympathetic effects of exercise training in heart failure. Am. J. Physiol. Heart Circ. Physiol. 298, H2032–2038 10.1152/ajpheart.00702.2009 20304818

[B91] Rengo G. Lymperopoulos A. Zincarelli C. Femminella G. Liccardo D. Pagano G. (2012a). Blockade of β-adrenoceptors restores the GRK2-mediated adrenal α(2) -adrenoceptor-catecholamine production axis in heart failure. Br. J. Pharmacol. 166, 2430–2440 10.1111/j.1476-5381.2012.01972.x 22519418 PMC3448904

[B92] Rengo G. Perrone-Filardi P. Femminella G. D. Liccardo D. Zincarelli C. de Lucia C. (2012b). Targeting the β-adrenergic receptor system through G-protein-coupled receptor kinase 2: a new paradigm for therapy and prognostic evaluation in heart failure: from bench to bedside. Circ. Heart Fail. 5, 385–391 10.1161/CIRCHEARTFAILURE.112.966895 22589366

[B93] Rengo G. Zincarelli C. Femminella G. D. Liccardo D. Pagano G. de Lucia C. (2012c). Myocardial β(2) -adrenoceptor gene delivery promotes coordinated cardiac adaptive remodelling and angiogenesis in heart failure. Br. J. Pharmacol. 166, 2348–2361 10.1111/j.1476-5381.2012.01954.x 22452704 PMC3448898

[B94] Rinaldi B. Corbi G. Boccuti S. Filippelli W. Rengo G. Leosco D. (2006). Exercise training affects age-induced changes in SOD and heat shock protein expression in rat heart. Exp. Gerontol. 41, 764–770 10.1016/j.exger.2006.05.008 16822632

[B95] Rush J. W. Denniss S. G. Graham D. A. (2005). Vascular nitric oxide and oxidative stress: determinants of endothelial adaptations to cardiovascular disease and to physical activity. Can. J. Appl. Physiol. 30, 442–474 10.1139/h05-133 16258183

[B96] Salazar N. C. Vallejos X. Siryk A. Rengo G. Cannavo A. Liccardo D. (2013). GRK2 blockade with betaARKct is essential for cardiac beta2-adrenergic receptor signaling towards increased contractility. Cell. Commun. Signal. 11, 64 10.1186/1478-811X-11-64 23984976 PMC3846709

[B97] Savvidou M. D. Vallance P. J. Nicolaides K. H. Hingorani A. D. (2001). Endothelial nitric oxide synthase gene polymorphism and maternal vascular adaptation to pregnancy. Hypertension. 38, 1289–1293 10.1161/hy1201.097305 11751705

[B98] Scherrer-Crosbie M. Ullrich R. Bloch K. D. Nakajima H. Nasseri B. Aretz H. T. (2001). Endothelial nitric oxide synthase limits left ventricular remodeling after myocardial infarction in mice. Circulation 104, 1286–1291 10.1161/hc3601.094298 11551881

[B99] Schwarz P. Diem R. Dun N. J. Förstermann U. (1995). Endogenous and exogenous nitric oxide inhibits norepinephrine release from rat heart sympathetic nerves. Circ Res. 77, 841–848 10.1161/01.RES.77.4.841 7554131

[B100] Selemidis S. Dusting G. J. Peshavariya H. Kemp-Harper B. K. Drummond G. R. (2007). Nitric oxide suppresses NADPH oxidase-dependent superoxide production by S-nitrosylation in human endothelial cells. Cardiovasc. Res. 75, 349–358 10.1016/j.cardiores.2007.03.030 17568572

[B101] Stary H. C. Chandler A. B. Glagov S. Guyton J. R. Insull W. Jr. Rosenfeld M. E. (1994). A definition of initial, fatty streak, and intermediate lesions of atherosclerosis. A report from the Committee on Vascular Lesions of the Council on Arteriosclerosis, American Heart Association. Circulation 89, 2462–2478 10.1161/01.CIR.89.5.2462 8181179

[B102] Sun D. Huang A. Zhao G. Bernstein R. Forfia P. Xu X. (2000). Reduced NO-dependent arteriolar dilation during the development of cardiomyopathy. Am. J. Physiol. Heart. Circ. Physiol. 278, H461–H468 10666076 10.1152/ajpheart.2000.278.2.H461

[B103] Taimeh Z. Loughran J. Birks E. J. Bolli R. (2013). Vascular endothelial growth factor in heart failure. Nat. Rev. Cardiol. 10, 519–530 10.1038/nrcardio.2013.94 23856679

[B104] Taylor M. R. Bristow M. R. (2004). The emerging pharmacogenomics of the beta-adrenergic receptors. Congest. Heart. Fail. 10, 281–288 10.1111/j.1527-5299.2004.02019.x 15591842

[B105] Tesauro M. Thompson W. C. Rogliani P. Qi L. Chaudhary P. P. Moss J. (2000). Intracellular processing of endothelial nitric oxide synthase isoforms associated with differences in severity of cardiopulmonary diseases: cleavage of proteins with aspartate vs. glutamate at position 298. Proc. Natl. Acad. Sci. U.S.A. 97, 2832–2835 10.1073/pnas.97.6.2832 10717002 PMC16015

[B106] Timmermann B. Mo R. Luft F. C. Gerdts E. Busjahn A. Omvik P. (1998). Beta-2 adrenoceptor genetic variation is associated with genetic predisposition to essential hypertension: the Bergen Blood Pressure Study. Kidney Int. 53, 1455–1460 10.1046/j.1523-1755.1998.00926.x 9607174

[B107] Toblli J. E. DiGennaro F. Giani J. F. Dominici F. P. (2012). Nebivolol: impact on cardiac and endothelial function and clinical utility. Vasc. Health Risk Manag. 8, 151–160 10.2147/VHRM.S20669 22454559 PMC3310359

[B108] Trochu J. N. Leblais V. Rautureau Y. Bévérelli F. Le Marec H. Berdeaux A. (1999). Beta 3-adrenoceptor stimulation induces vasorelaxation mediated essentially by endothelium-derived nitric oxide in rat thoracic aorta. Br. J. Pharmacol. 128, 69–76 10.1038/sj.bjp.0702797 10498836 PMC1571624

[B109] Troost R. Schwedhelm E. Rojczyk S. Tsikas D. Frölich J. C. (2000). Nebivolol decreases systemic oxidative stress in healthy volunteers. Br. J. Clin. Pharmacol. 50, 377–379 10.1046/j.1365-2125.2000.00258.x 11012562 PMC2014994

[B110] Tulis D. A. (2008). Novel therapies for cyclic GMP control of vascular smooth muscle growth. Am. J. Ther. 15, 551–564 10.1097/MJT.0b013e318140052f 19127140 PMC2677189

[B111] Tzemos N. Lim P. O. MacDonald T. M. (2001). Nebivolol reverses endothelial dysfunction in essential hypertension: a randomized, double-blind, crossover study. Circulation 104, 511–514 10.1161/hc3001.094207 11479245

[B112] Vanhoutte P. M. Gao Y. (2013). Beta blockers, nitric oxide, and cardiovascular disease. Curr. Opin. Pharmacol. 13, 265–273 10.1016/j.coph.2012.12.002 23294896

[B113] Vecchione C. Aretini A. Marino G. Bettarini U. Poulet R. Maffei A. (2006). Selective Rac-1 inhibition protects from diabetes-induced vascular injury. Circ. Res. 98, 218–225 10.1161/01.RES.0000200440.18768.30 16357302

[B114] Wiemer G. Itter G. Malinski T. Linz W. (2001). Decreased nitric oxide availability in normotensive and hypertensive rats with failing hearts after myocardial infarction. Hypertension 38, 1367–1371 10.1161/hy1101.096115 11751719

[B115] Wojciechowski D. Papademetriou V. (2008). Beta-blockers in the management of hypertension: focus on nebivolol. Expert. Rev. Cardiovasc. Ther. 6, 471–479 10.1586/14779072.6.4.471 18402537

[B116] Wu K. K. (2002). Regulation of endothelial nitric oxide synthase activity and gene expression. Ann. N.Y. Acad. Sci. 962, 122–130 10.1111/j.1749-6632.2002.tb04062.x 12076969

[B117] Wyatt A. W. Steinert J. R. Wheeler-Jones C. P. Morgan A. J. Sugden D. Pearson J. D. (2002). Early activation of the p42/p44MAPK pathway mediates adenosine-induced nitric oxide production in human endothelial cells: a novel calcium-insensitive mechanism. FASEB J. 16, 1584–1594 10.1096/fj.01-0125com 12374781

[B118] Yang H. T. Prior B. M. Lloyd P. G. Taylor J. C. Li Z. Laughlin M. H. (2008). Training-induced vascular adaptations to ischemic muscle. J. Physiol. Pharmacol. 59(Suppl. 7), 57–70 19258657 PMC2654575

[B119] Yetik-Anacak G. Catravas J. D. (2006). Nitric oxide and the endothelium: history and impact on cardiovascular disease. Vascul. Pharmacol. 45, 268–276 10.1016/j.vph.2006.08.002 17052961

[B120] Zhang X. Lynch A. I. Davis B. R. Ford C. E. Boerwinkle E. Eckfeldt J. H. (2012). Pharmacogenetic association of NOS3 variants with cardiovascular disease in patients with hypertension: the GenHAT study. PLoS ONE 7:e34217 10.1371/journal.pone.0034217 22470539 PMC3314599

